# Supplementary material for: Old Tricks, New Opportunities: How Companies Violate the International Code of Marketing of Breast-Milk Substitutes and Undermine Maternal and Child Health during the COVID-19 Pandemic
Source: Int J Environ Res Public Health. 2021 Mar 1;18(5):2381. doi: 10.3390/ijerph18052381 (PMC7967752; doi:10.3390/ijerph18052381)
Supplement: Supplementary file 1 [file ijerph-18-02381-s001.zip › Supplementary caption.docx]

| Figure S1 | Label of Colosbaby, manufactured by VitaDairy in Vietnam. The text on label claims to “boost immune system, prevent respiratory and digestive infections caused by viruses and bacteria” (translated from Vietnamese). |
| --- | --- |
| Figure S2 | Screenshot of an article with a banner “Wuhan add oil (keep it up), united with a single will strong like a fortress, we can get through the challenges together” (translated from Chinese). It appeared in a news portal in China, detailing donations and “nutritional support” from FrieslandCampina and its partner China Women's Development Foundation. |
| Figure S3 | Screenshot of Feihe’s donation campaign from the National Business Daily news portal in China. In partnership with Chinese Red Cross Foundation, the company donated medical equipment to hospitals. The e-poster featured the tagline “Hang in there Wuhan. China add oil (keep it up)” (translated from Chinese). |
| Figure S4 | Screenshot of a news article about Nestle’s donation of medical equipment to the Ministry of Health in Burkina Faso. |
| Figure S5 | In partnership with OnDoctor App, the nutrition vlog featured an influencer nutritionist, sponsored by Dugro 3 (Danone) in Myanmar (Facebook). |
| Figure S6 | Dugro 3 (Danone) banner advertisement appearing in the Myanmar Parenting Group on Facebook. |
| Figure S7 | Pigeon India #StandbyNursingMoms campaign. “We know breastfeeding is not easy but it’s definitely worth the dedication!” appeared in one of the posts on Facebook. |
| Figure S8 | Information of a webinar on COVID-19 and breastfeeding from Medela US website. It states “all major organizations agree that the provisions of breastmilk is important”, but omitted breastfeeding is itself. |
| Figure S9 | The Hi-Family Club Facebook page (sponsored by Nutricia, Danone) in Laos posted a video that cautioned parents to “check what each color of (breast)milk indicates and whether it is harmful to the baby” (translated from Lao). |
| Figure S10 | Screenshot from Gerber (Nestle) US website, offering discounts on formula that are linked to COVID-19. |
| Figure S11 | Similac (Abbott) US website that addresses the difficult times of COVID-19 and offering special deals and gifts. |
| Figure S12 | Screenshot of an article on the Healthy Newborn Network reporting on how BMS industry approach health workers. |
