# Supplementary figures and images for: Old Tricks, New Opportunities: How Companies Violate the International Code of Marketing of Breast-Milk Substitutes and Undermine Maternal and Child Health during the COVID-19 Pandemic
Source: Int J Environ Res Public Health. 2021 Mar 1;18(5):2381. doi: 10.3390/ijerph18052381 (PMC7967752; doi:10.3390/ijerph18052381)

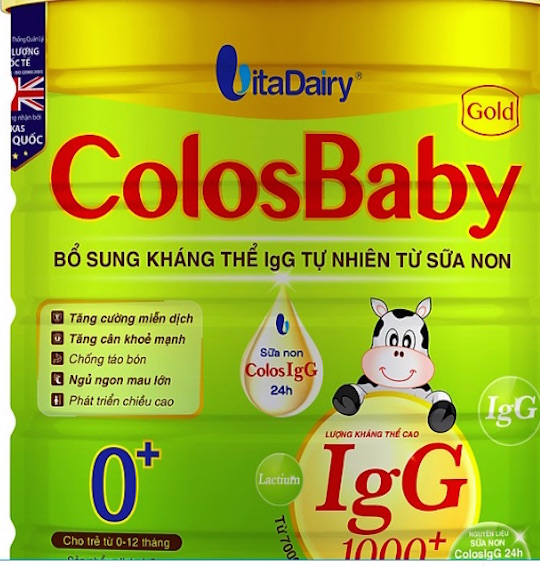

Supplement: Supplementary file 1 [file ijerph-18-02381-s001.zip › Figure S1.jpeg]

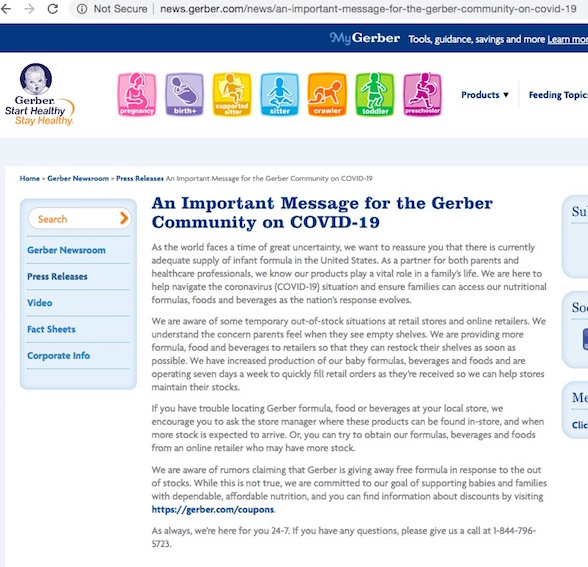

Supplement: Supplementary file 1 [file ijerph-18-02381-s001.zip › Figure S10.jpeg]

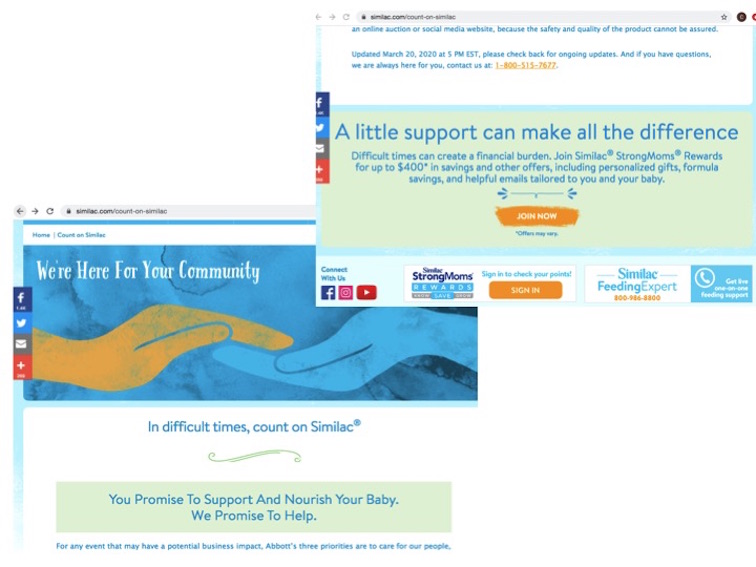

Supplement: Supplementary file 1 [file ijerph-18-02381-s001.zip › Figure S11.jpeg]

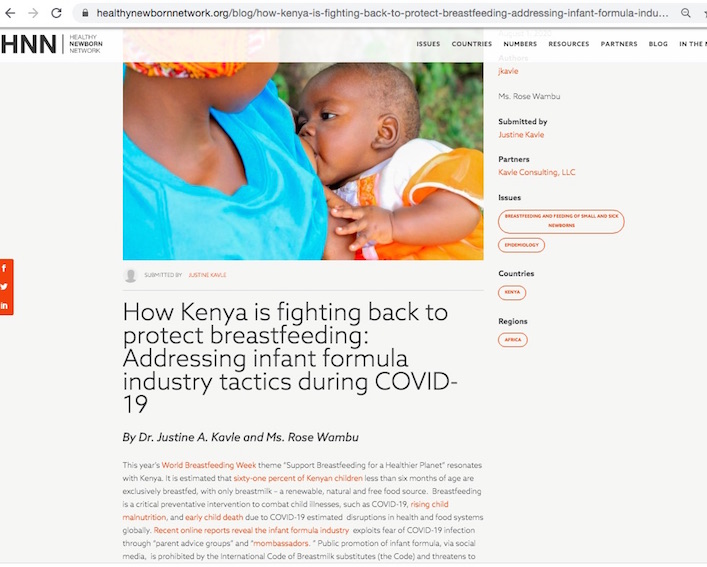

Supplement: Supplementary file 1 [file ijerph-18-02381-s001.zip › Figure S12.jpeg]

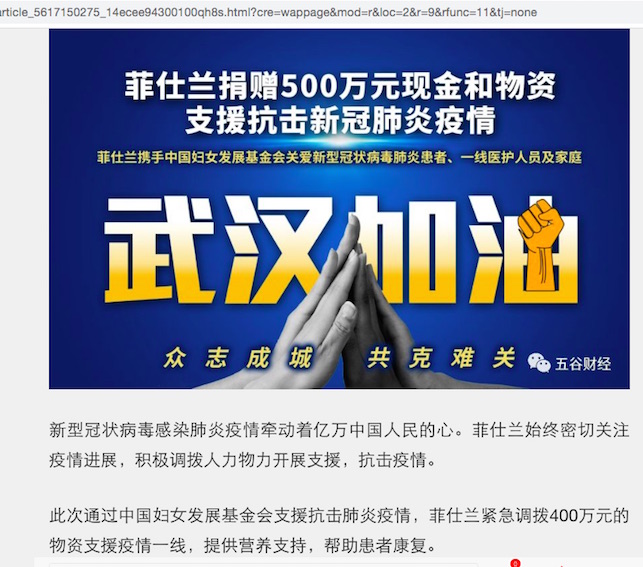

Supplement: Supplementary file 1 [file ijerph-18-02381-s001.zip › Figure S2.jpeg]

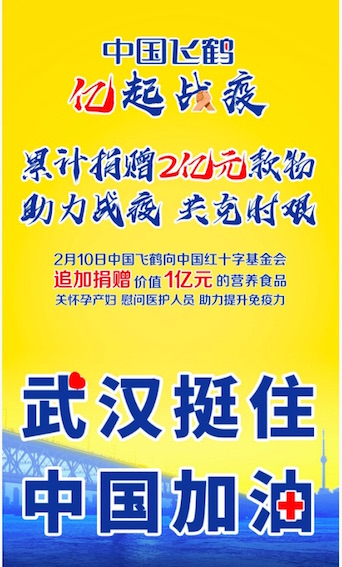

Supplement: Supplementary file 1 [file ijerph-18-02381-s001.zip › Figure S3.jpeg]

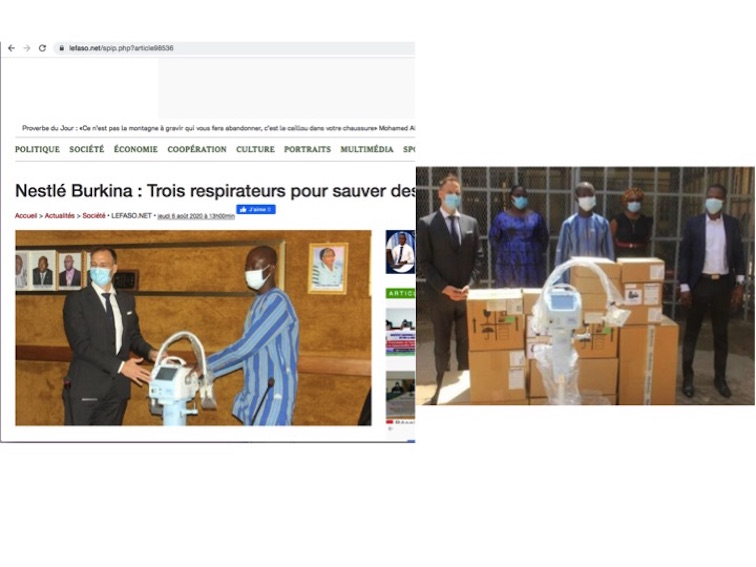

Supplement: Supplementary file 1 [file ijerph-18-02381-s001.zip › Figure S4.jpeg]

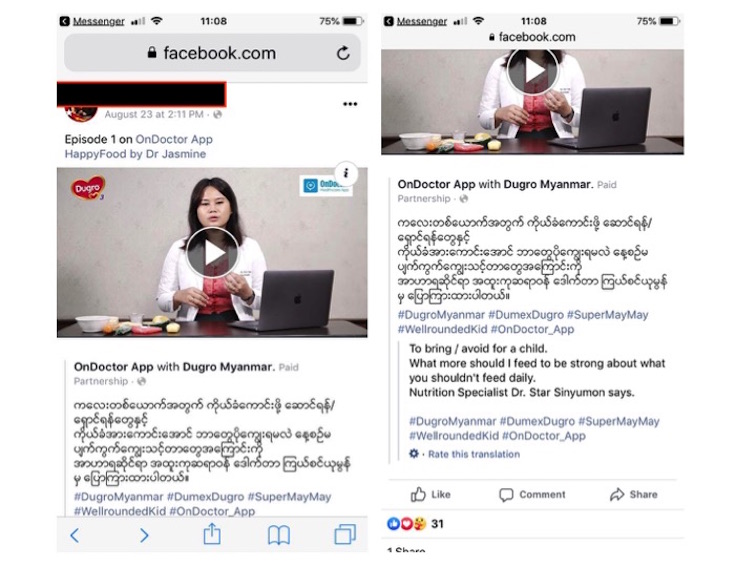

Supplement: Supplementary file 1 [file ijerph-18-02381-s001.zip › Figure S5.jpeg]

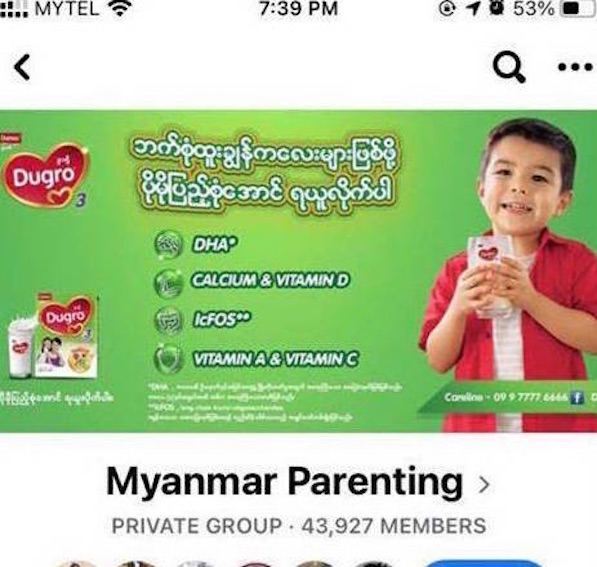

Supplement: Supplementary file 1 [file ijerph-18-02381-s001.zip › Figure S6.jpeg]

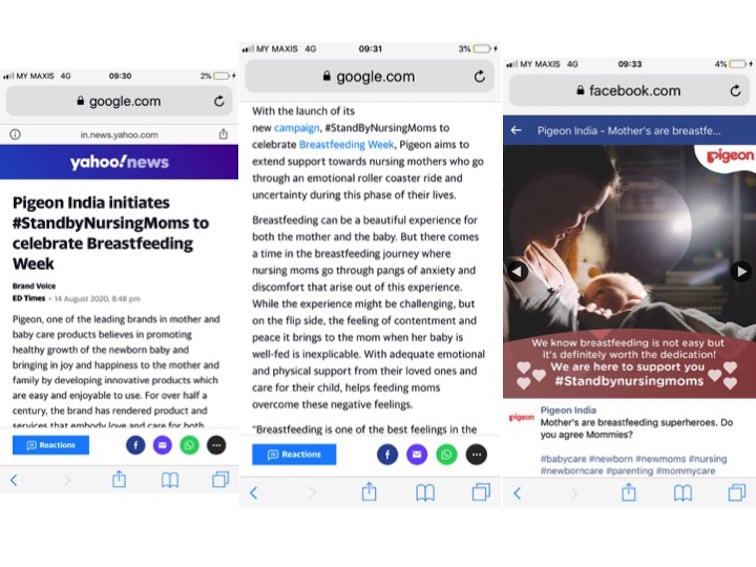

Supplement: Supplementary file 1 [file ijerph-18-02381-s001.zip › Figure S7 .jpeg]

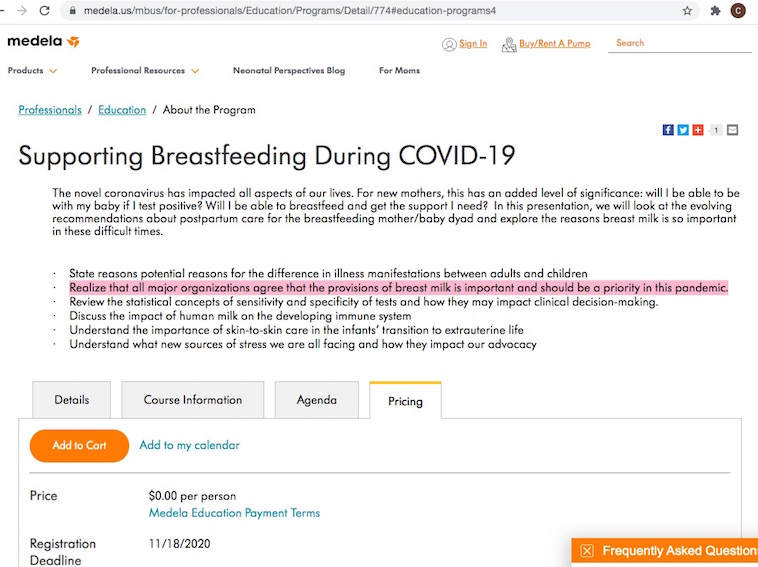

Supplement: Supplementary file 1 [file ijerph-18-02381-s001.zip › Figure S8.jpeg]

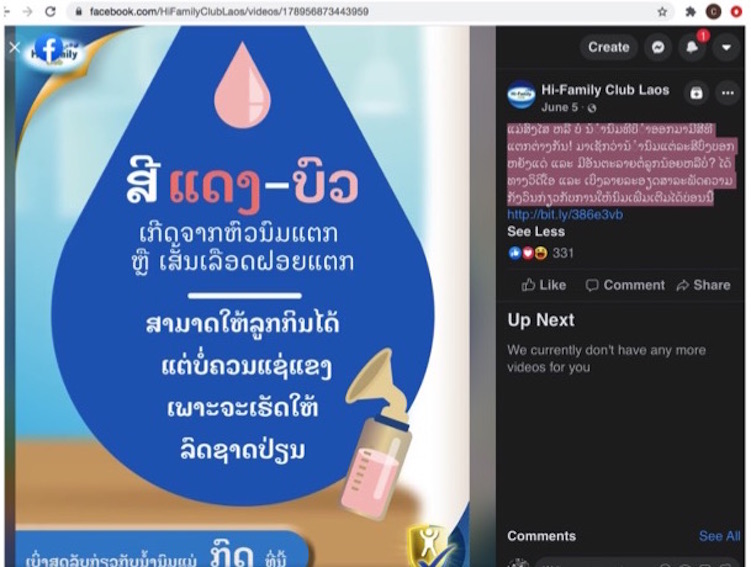

Supplement: Supplementary file 1 [file ijerph-18-02381-s001.zip › Figure S9.jpeg]
